# Exploration of the Existence Forms and Patterns of Dissolved Oxygen Molecules in Water

**DOI:** 10.1007/s40820-024-01427-z

**Published:** 2024-06-04

**Authors:** Hewei Yuan, Yaozhong Zhang, Xiaolu Huang, Xiwu Zhang, Jinjin Li, Yufeng Huang, Kun Li, Haotian Weng, Yang Xu, Yafei Zhang

**Affiliations:** 1 Key Laboratory of Thin Film and Microfabrication (Ministry of Education), Department of Micro/Nano Electronics, School of Electronic Information and Electrical Engineering, Shanghai Jiao Tong University, Shanghai, 200240 People’s Republic of China; 2 School of Materials Science and Engineering, Shanghai Jiao Tong University, Shanghai, 200240 People’s Republic of China; 3 Jinduo Yuchen Water Environment Engineering Co., Ltd, Shanghai, 201702 People’s Republic of China

**Keywords:** Water clusters, Dissolved oxygen, ^17^O NMR, Molecular dynamics simulation

## Abstract

The structure of liquid water is primarily composed of three-dimensional networks of water clusters formed by hydrogen bonds, and dissolved oxygen is one of the most important indicators for assessing water quality. In this work, distilled water with different concentration of dissolved oxygen were prepared, and a clear negative correlation between the size of water clusters and dissolved oxygen concentration was observed. Besides, a phenomenon of rapid absorption and release of oxygen at the water interfaces was unveiled, suggesting that oxygen molecules predominantly exist at the interfaces of water clusters. Oxygen molecules can move rapidly through the interfaces among water clusters, allowing dissolved oxygen to quickly reach a saturation level at certain partial pressure of oxygen and temperature. Further exploration into the mechanism by molecular dynamics simulations of oxygen and water clusters found that oxygen molecules can only exist stably at the interfaces among water clusters. A semi-empirical formula relating the average number of water molecules in a cluster (*n*) to ^17^O NMR half-peak width (*W*) was summarized: *n* = 0.1 *W* + 0.85. These findings provide a foundation for exploring the structure and properties of water.

## Highlights

- A clear negative correlation between the size of water clusters and the concentration of dissolved oxygen was observed. This implied that smaller clusters water exhibits higher concentrations of dissolved oxygen.
- Oxygen molecules are primarily existed at the surfaces or interfaces of water clusters and can rapidly traverse the gas liquid interface.
- A semi empirical formula relating the average number of water molecules in a cluster to ^17^O NMR half peak width was derived, demonstrating an approximate lin ear relationship.

## Graphical abstract

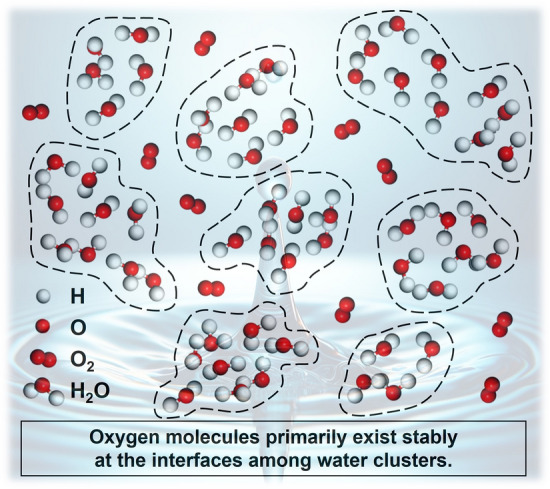

## Introduction

**Fig. 1:**
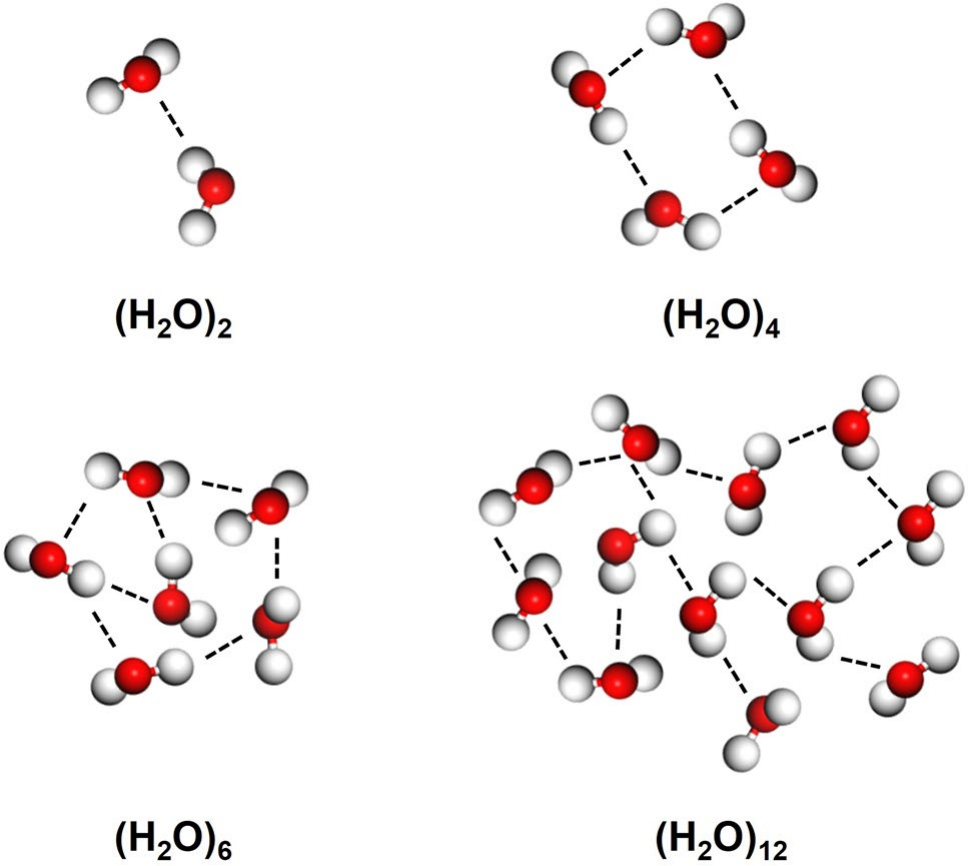
The structure of water clusters ((H_2_O)_n_, where *n* = 2, 4, 6, and 12)

Water is one of the most essential substances in the world for human life. It has some special properties like high melting point, boiling point, and surface tension. These phenomena can be well explained by the cluster model of liquid water structure [1, 2], which assumes that water consists of continuously distributed cluster sizes and has been widely studied for its central role in life, geophysics, biochemistry, etc. [3]. H_2_O is a polar molecule, meaning it has a partial positive charge near the hydrogen atoms and a partial negative charge near the oxygen atom. A hydrogen bond forms when the hydrogen atom from one water molecule is attracted to the oxygen atom of another water molecule. Thus, as water molecules come into proximity, they can form hydrogen bonds with neighboring molecules. Because each water molecule can potentially form up to four hydrogen bonds, complex networks or clusters of molecules can form. At different conditions, the number of water molecules contained in a cluster may vary, resulting in various sizes of water clusters. For example, the size of water clusters (*n*) may be 2, 4, 6, and 12, or even larger, as shown in Fig. 1 [4–7]. This structural arrangement significantly helps to maintain the liquid state of water. Therefore, the study of small water clusters (SWCs) is not only beneficial for revealing the lining dynamics in water droplets, but also has practical value for applications in various fields, such as the conformation of biological macromolecules (especially proteins and nucleic acids), suitable water injection in ultra-low permeability reservoirs, and the dissolution of ions in minerals [8–13]. As a medium for biological processes, water clusters have a dramatic impact on the structure, function, and cellular activity of almost all biomolecules [14–16]. The structural complexity of water makes it exhibit several unique properties that have a significant impact on the formation of living organisms and environmental conditions. A deep understanding of the structure of water contributes to a comprehensive understanding of its properties and functions, which is the basis and prerequisite for research in many fields.

Over the past 100 years, the structure of liquid water has been intensively studied in different fields such as physics, chemistry, and physical chemistry [17]. So far, theoretical simulations and experimental techniques have been used to study the properties of water clusters, particularly the distribution of water cluster sizes [18–24]. Among them, computational methods have been widely used to infer the structure and thermodynamic properties of water clusters [25–29], and simulation results show that a certain water cluster (e.g., (H_2_O)_n_, where *n* = 3, 4, and 6) exhibits a relatively stable structure [15]. However, the complex potential energy faces inherent in hydrogen bond clusters pose an obstacle to a water model that provides an accurate description [11]. In addition, the ability to study large water clusters ((H_2_O)_n_, *n* > 10) is limited due to increased computational demands. For small water clusters, the quasi-planar ring structure is more suitable for water trimer to pentamer structure, with each monomer being both proton donor and acceptor, while the cage rather than ring structure is more suitable for water hexamer. For aqueous heptamers and larger clusters, the simulations predict their tendency to evolve into three-dimensional structures [30–33]. This ongoing research reveals the complexity of the water substructure and has profound implications for multiple scientific fields.

In addition to simulations, many experimental techniques have also been applied to investigate the structure of water molecules, including inelastic neutron scattering [34], infrared spectroscopy [35], Raman spectroscopy [36], X-ray diffraction [27], neutron diffraction [37], and nuclear magnetic resonance (NMR) spectroscopy [38], etc. For example, in 1977, Dyke et al. [39] observed structural information about water dimers and matched it to a rigid rotor model by means of electric resonance spectroscopy. In 1992, Pugliano et al. [40] utilized the vibration–rotation tunneling (VRT) bands of the absorption spectrum via tunable far-infrared laser to detect the structure of water trimer clusters, sparking interest in confirming theoretical predictions of larger water molecular cluster structures. Mass spectrometry is a powerful tool for determining the size distribution of water molecular clusters [41]. For example, in 1989, Yeh et al. [42] detected small protonated water clusters (H_2_O)_n_ H^+^ (*n* = 1–4) in the gas phase through a series in-mass selection spectroscopy technique. In 2010, Conlan et al. [43] simultaneously detected two series of cluster ions ((H_2_O)_n_ H^+^ and (H_2_O)_n_) in water ice by secondary ion mass spectrometry (SIMS) equipped with Au^+^, Au^3+^, and C_60_^+^ as the dominant ion. In 2014, Servage et al. [44] investigated the structural transformation of these protonated water clusters by low temperature (80 K) ion migration-mass spectrometry (cryo-IM-MS). However, most of these studies are limited to the analysis of water clusters in the gas phase or water ice because of the techniques used require a vacuum environment, and directly testing the size distribution of water clusters in ambient aqueous solutions remains a challenge. To this end, in 2016, Kusakari et al. [45] developed an environmental SIMS incorporating ion probes for water clusters detection at room temperature, despite the fact that maintaining molecular integrity remains a major challenge because of the increased fragmentation of water clusters due to high ion energies.

NMR is one of the most effective methods for studying the structure of water in liquid and aqueous solutions. Results of the ^17^O NMR are considered useful for probing the structure, dynamics, and intermolecular and intramolecular hydrogen bonding effects of liquid water, such as the half-peak width of the spectrum. Although water is non-magnetic, its nuclei can sense external magnetic fields and produce weak reactions. The hydrogen nucleus in the water rotates like the Earth, acting like a small magnet. When a magnetic field is applied externally, these hydrogen nuclei rotate in the direction of the magnetic field and absorb the wave number of electromagnetic waves that match the rotation speed while encountering electromagnetic waves. The linewidth of the NMR spectrum of liquid water is mainly affected by the transverse relaxation process. Since the transverse relaxation process involves the exchange of spin states between adjacent nuclei, and the larger the size of water clusters, the faster the exchange of spin states between oxygen or hydrogen nuclei and adjacent nuclei, the shorter the time required for the sample to return to equilibrium, resulting in a wider spectral line [46–48]. As a result, while using NMR to analyze the distribution of hydrogen nuclei in water molecules, the size of water molecular clusters can be concluded as well [49, 50]. Also, it is possible to understand how fast these water molecules are moving: the slower the molecules motion, the larger the molecular size, the shorter the relaxation time, and the wider the half-peak width is [51]. This correlation between NMR frequency and molecular cluster size reveals important dynamic behavior at the molecular level of water [52, 53].

The structure of water is related to hydrogen bonding and can be affected by chemical and physical factors such as ions, temperature, and pressure. A study investigating the relationship between ^17^O NMR half-peak width and water clusters showed that the half-peak width decreased as the temperature increased [54]. Ions in water can also change the size and distribution of water clusters by affecting hydrogen bonds [55]. For example, Walrafen et al. [36] observed that the Raman strength of liquid water decreased with an increasing temperature, while Ruhua et al. found a linear relationship between NMR chemical shift of water and ion concentration [56]. In addition, several methods can be applied to change the structure of water clusters, such as external electric field [57], magnetic field [58–60], microwave radiation [61], and laser radiation [62]. For example, Rai et al. [57] used density functional theory to study the structure of water clusters induced under a uniform static external electric field and found that as the field strength increased, the number of hydrogen bonds decreased. Yap et al. [60] found that magnetic effect improved pH and concentration of dissolved oxygen, decreased oxidation–reduction potential (ORP), strengthened intermolecular hydrogen bonding of water molecules and promoted smaller water clusters formation. A research by Fesenko et al. [61] showed that microwave radiation changed the state of water. These studies illustrate the complex interaction of external influences on the water structure, highlighting the potential for targeted manipulation of its structural properties.

Facing the challenges of increasing water scarcity and serious water pollution, research on water sciences has become increasingly important [63, 64]. Dissolved oxygen (DO) is another important factor in the research of water. Scientists currently believe that oxygen molecules exist in water in a dissolved state, called dissolved oxygen. Dissolved oxygen is one of the most important physical and chemical indexes in the structure of water, and its concentration has an important effect on the growth and reproduction of aquatic organisms and the metabolism and transformation of nutrients in water [65]. Therefore, an in-depth study of the state and mechanism of DO is of great significance for understanding the characteristics of aquatic environments and the stability of aquatic ecosystems. Firstly, the concentration of dissolved oxygen is one of the most important indicators to judge the degree of eutrophication and water quality. Low concentration of dissolved oxygen in water may lead to asphyxiation and death of aquatic organisms, and even cause water eutrophication and other problems [66]. Therefore, the measurement and monitoring of dissolved oxygen can provide an important judgement for water resource management and protection. Secondly, the state and mechanism of dissolved oxygen can also reveal the process of material transformation in water ecosystems. The interaction and transformation process between organic matter, inorganic matter and microorganism in water is closely related to dissolved-oxygen. For example, the degradation of organic matter and the oxidation of inorganic matter require a large amount of oxygen, and the growth and metabolism of microorganisms also require an adequate supply of oxygen. Therefore, a thorough investigation into the mechanisms of dissolved oxygen in these processes can help us better understand the ecosystems in water. Finally, with the aggravation of environmental pollution and the impact of global climate change, the concentration and distribution of dissolved oxygen in water are facing more and more challenges [67, 68]. To deal with these challenges, we can better predict and evaluate the varying trends of water environment by in-depth research on the state and mechanism of dissolved oxygen and propose effective management and protection strategies to promote the healthy development of water ecosystems.

Therefore, it is of great significance to conduct theoretical research on dissolved oxygen in water. However, the research of the existence forms and patterns of dissolved oxygen molecules in water remains insufficiently now, and the theoretical and practical understanding of the improvement of dissolved oxygen enhancement is still inadequate. Based on this, in this work, we used a series of distilled water with different concentration of dissolved oxygen and evaluated the size of water clusters of all the samples by ^17^O NMR spectroscopy. The results showed that the concentration of dissolved oxygen in the water exhibited a significant negative correlation with the size of water molecular clusters. Based on this, we put forward the view that oxygen molecules mainly exist at the surface or interface of water molecular clusters. The smaller the size of water clusters, the more interfacial voids, the more oxygen molecules can be accommodated, and the higher the concentration of dissolved oxygen of the water. Also, we verify the theory by calculating the molecular dynamics model. In addition, we carried out case studies on five water samples of small clusters water, purified water, deionized water, distilled water, and rainwater, and found that the relationship between dissolved oxygen and water clusters showed the same rule, which provided a theoretical basis for the increasement of dissolved oxygen in water.

## Experimental Section

The distilled water used in the experiments was obtained through a water distiller by Fuyao (China, Model K40103) in the laboratory. Oxygen was sourced from Shanghai Wetry Standard Gas Analysis Technology Co., Ltd. (Shanghai, China), ensuring a consistent and reliable supply of high-quality oxygen for our research.

The experimental operations were conducted in a 500 mL three-neck flask, with one neck connected to a jet pump for the pre-extraction of gases from the flask to remove excess gases, ensuring all water samples maintain the same initial state. The second neck was connected to an oxygen pipeline, serving as the channel for introducing oxygen into the water samples. The third neck was connected to a dissolved oxygen meter or other relevant testing equipment. To ensure consistency among the water samples and eliminate interference from pre-existing gases, the gases in the flask were pre-extracted by the jet pump for 60 min after filling the flask with the experimental water samples. Subsequently, oxygen was slowly introduced into the water at a flow rate of 50 sccm. During this process, the tube connected to the pump kept opened, allowing the excess oxygen that could not dissolve in the water to be naturally expelled. At this stage, the concentration of dissolved oxygen in the water was continuously monitored and recorded. Once the concentration of dissolved oxygen was stabilized, the system was considered to reach an equilibrium state, and the stable dissolved oxygen values recorded in various water samples represented their saturation concentration under the experimental conditions. After 60 min, the oxygen supply was turned off, and the process of naturally decreasing of the concentration of dissolved oxygen was continuously recorded as well. External conditions such as time and temperature were strictly controlled and kept consistent across different water samples to eliminate any form of interference, thereby ensuring the reliability and accuracy of the experimental results.

After the concentrations of dissolved oxygen of water samples reached given values, respectively, they were transferred to NMR tubes and sealed quickly. In previous experiments, we found that the dissolved oxygen in water could remain almost unchanged for a long period of time when sealed in the containers. Moreover, the water samples were sealed within the NMR tubes throughout the NMR testing process, thereby the respective concentrations of dissolved oxygen could be maintained.

The measurement of dissolved oxygen was performed utilizing a dissolved oxygen meter by SmartSensor (China, model AR8406) with a dissolved oxygen measurement range of 0 ~ 30 mg L^−1^, resolution of 0.1 mg L^−1^, and accuracy of ± 3%. A 100% saturation dissolved oxygen calibration was performed before testing to ensure the accuracy and reliability of data collection. The characteristics of water clusters were analyzed utilizing a NMR equipment by Bruker (Germany, model Avance Neo) with an operating frequency of 400.13 MHz, main magnetic field of 9.4 Tesla (superconducting) and testing temperature of 298 K. ^17^O NMR tests were performed with chemical shifts measured relative to the standard of pure nitromethane.

The calculations were conducted by the Vienna Ab Initio Simulation Package, with the exchange–correlation functional set to PW91. The Projector Augmented-Wave (PAW) method was employed to treat the interactions between ions/electrons [12, 69]. The plane-wave cutoff energy was set to 400 eV. The structure of the water clusters was placed in a cubic cell with lattice parameters of 20 Å × 20 Å × 20 Å or 40 Å × 20 Å × 20 Å.

## Results and Discussion

### Relationship Between Dissolved Oxygen and Cluster Structure

**Fig. 2:**
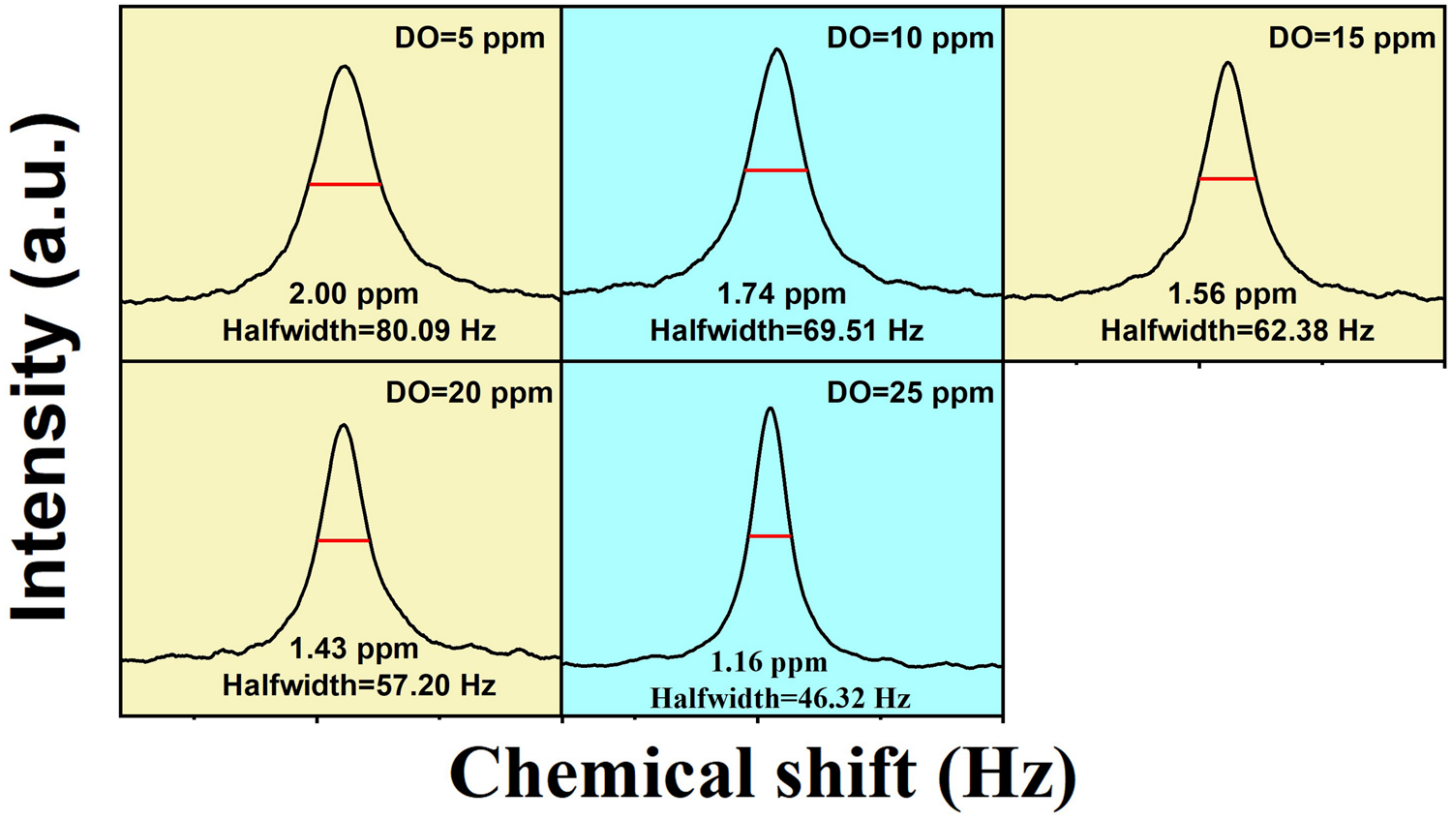
^17^O nuclear magnetic resonance spectra of distilled water with varying concentration of dissolved oxygen

To analyze the relationship between the concentration of dissolved oxygen and the structure of water clusters, water samples of various concentration was employed for testing to analyze their relationship. Distilled water was selected to eliminate the impact of impurities and ions. Oxygen was firstly introduced into five distilled water samples for varying durations to obtain different concentration of water samples, and then the samples were performed ^17^O NMR tests promptly. As can be seen in Fig. 2, a significant negative correlation between the saturated concentration of dissolved oxygen and the size of water clusters could be observed: the lower the half-peak width of the NMR spectrum, the smaller the water clusters [50], and consequently, the higher the concentration of dissolved oxygen.

According to the literature [70], oxygen molecules tend to exist in a cavity in liquid water upon dissolving into water. We proposed a hypothesis that as the size of water clusters decreases, the number of inter-cluster interstitial volume increases, and the volume of these cavities expands as well. As a result, a higher saturated concentration of dissolved oxygen can be achieved due to the oxygen molecules are inclined to occupy the spaces among water clusters.

### Simulations of Oxygen in Relation to Water Clusters Positions

**Fig. 3:**
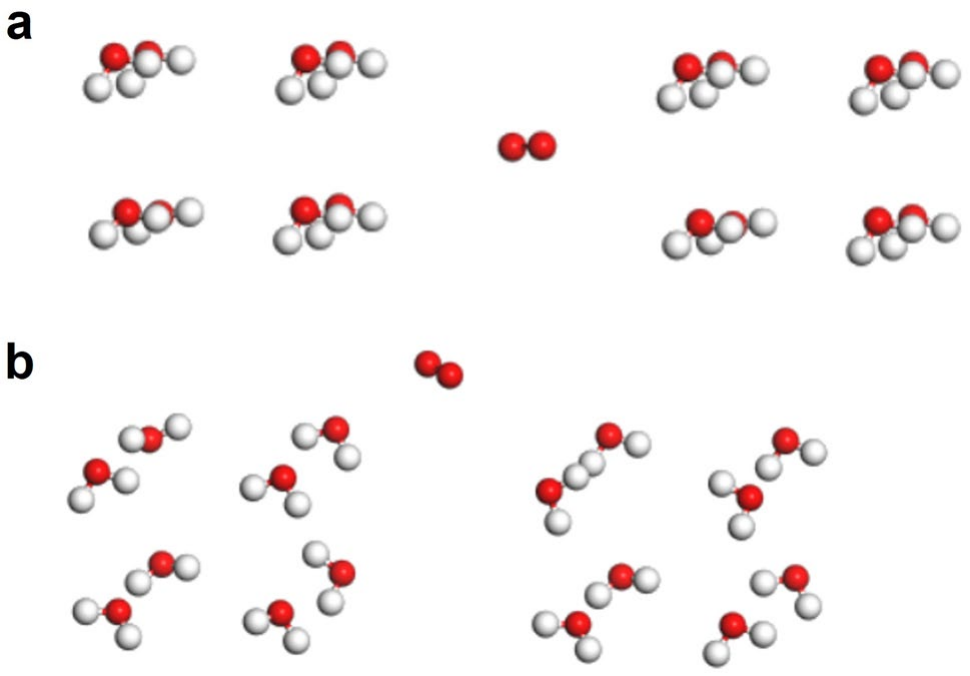
Simulations of the positional relationship between an oxygen molecule and a water cluster (*n* = 16). Situation of oxygen **a** inside water cluster and **b** outside water cluster

In response to the above assumption, simulations of the positional relationship states among oxygen molecules and water clusters were performed as a potential method for verification. Firstly, for the case of a single water cluster (*n* = 16, combined by two representative water clusters of *n* = 8) and one oxygen molecule as shown in Fig. 3, the water cluster gradually deformed and could not maintain a stable structure when the oxygen molecule was inside the water cluster (Fig. 3a). Despite changing the distance among water molecules several times to adjust the configuration of the clusters, the system still could not remain equilibrium. This result showed that the system was unstable when the oxygen molecule was inside. On the contrary, when the oxygen molecule was at the surface of the water cluster (*n* = 16) as shown in Fig. 3b, the calculation could converge normally, and the structure and energy (−235.97 eV) could retain stable over a long period of time, which indicated that the activity of the oxygen molecule at the surface of the water cluster was higher than that of inside the cluster, and the system was more stable. These results showed that oxygen molecules tended to reside at the surface of water clusters rather than inside them.

**Fig. 4:**
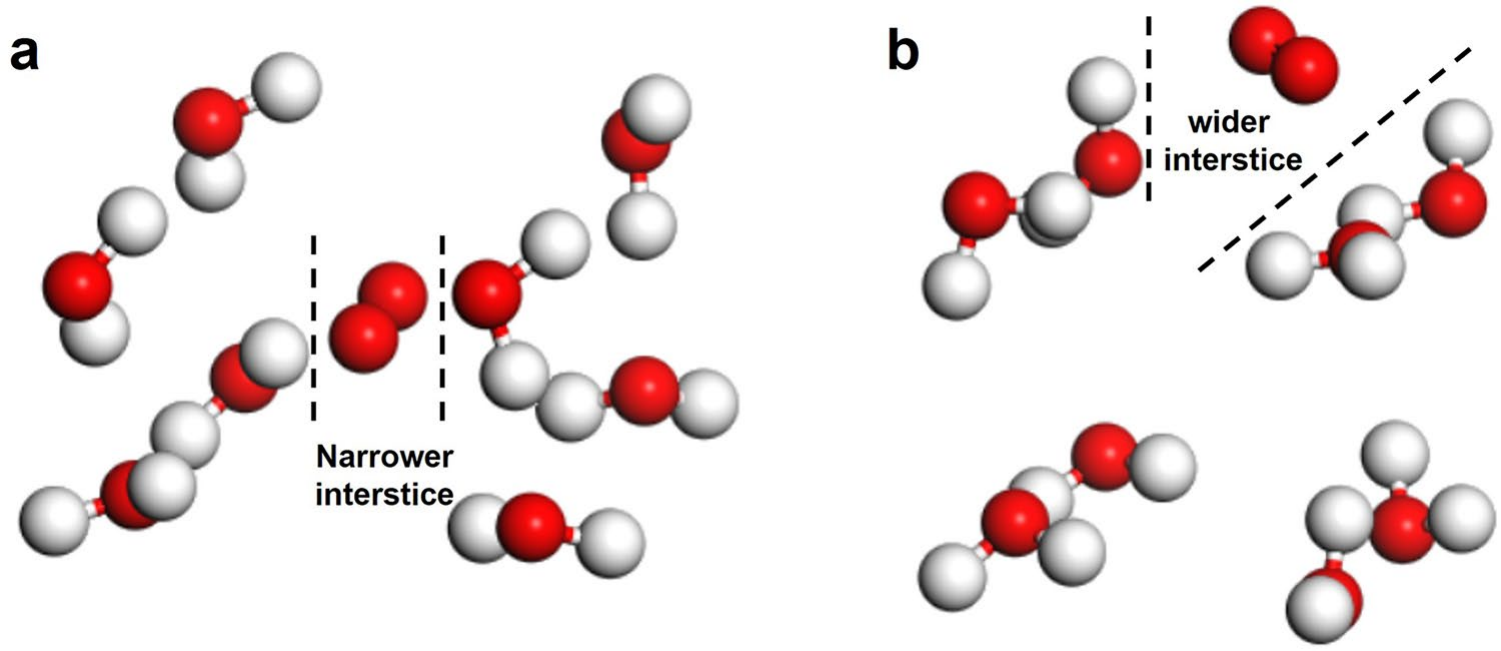
Simulations of the state of oxygen molecule in different interstices of water clusters (*n* = 2). Situation of **a** narrow interstice and **b** wide interstice

Furthermore, we calculated the situation of oxygen in different interstices of water clusters (*n* = 2, for simplifying the calculation). The energy of the system was -123.70 eV while the oxygen molecule was in a narrow interstice as shown in Fig. 4a, whereas the energy of the system was reduced to −122.95 eV while the oxygen molecule was in a wide interstice as shown in Fig. 4b. At the latter situation, the energy was lower, and the system was more stable, which indicated that the wider the interstices, the more favorable to the existence of oxygen molecules. As a result, small clusters water with smaller size of clusters, higher activity and wider interstices are more appealing to oxygen attachment, and thus has higher saturated concentration of dissolved oxygen. This was consistent with the hypothesis proposed in Sect. 3.1.

### Relationship Between Water Clusters and ^17^O Nnuclear Magnetic Resonance Half-Peak Width

Water clusters are composed of several water molecules connected by hydrogen bonds to form a network structure. Although the number of water molecules contained in each cluster may vary, ranging from small clusters of 2 to 10 molecules to larger clusters of 10 to 40 or even more, and the clusters are in dynamic change, for a given water, the average number of water clusters in a certain period is determined. The size of water clusters directly affects the physical, chemical, and thermodynamic properties of water [1, 71, 72]. Therefore, it is necessary to analyze the size of water clusters, and the ^17^O NMR half-peak width represents the average cluster number of water molecules [52, 53].

**Fig. 5:**
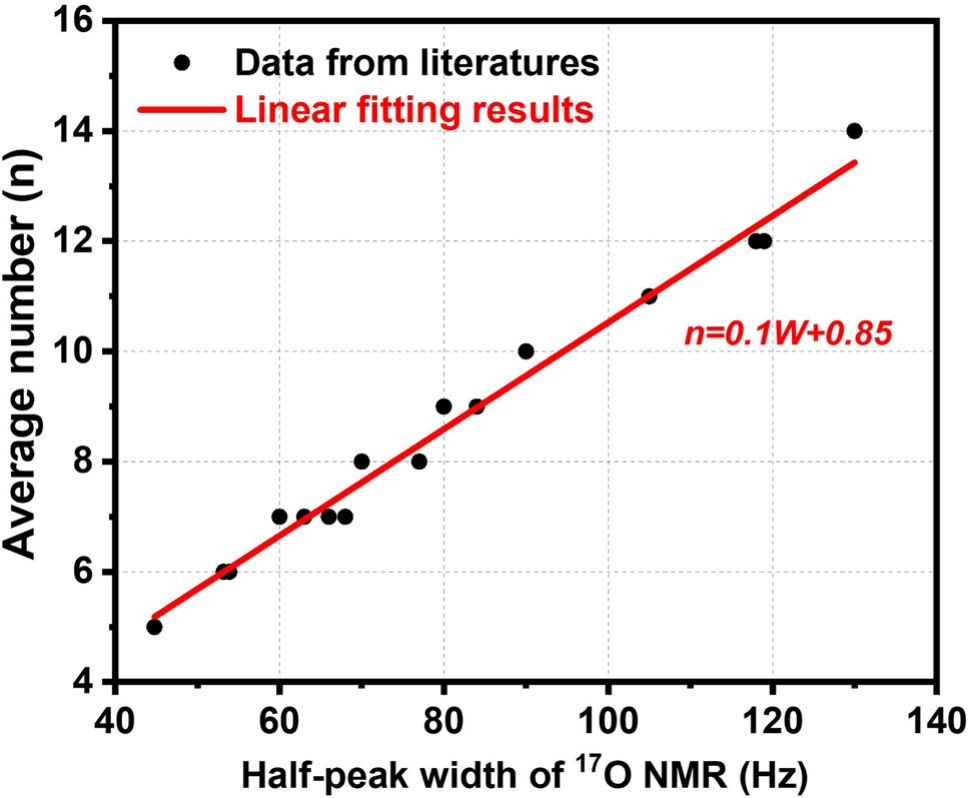
Correspondence and the linear fitting curve between the average number of water molecules in a cluster and ^17^O NMR half-peak width

We compiled data on the relationship between NMR half-peak width and the average number of water molecules in a cluster tested by various researchers, and the results are presented in Table 1 and Fig. 5. A nearly linear positive correlation between NMR half-peak width and the average number of water molecules in a cluster was observed. Similarly, Troganis et al. [73] found that the mass of different sized protein molecules was linear to the half-peak width of ^14^N and ^17^O NMR, which aligned with our findings. Based on these data, a linear fitting was performed, and a semi-empirical formula relating the average number of water molecules in a cluster to ^17^O NMR half-peak width was obtained:

1 $$ {{n}} = 0.{{1\;  W}} + 0.{85} $$

where *n* represents the average number of water molecules in a cluster, and *W* represents the half-peak width in the ^17^O NMR spectra (Hz).

**Table 1:** Data from literatures on ^17^O NMR half-peak width and the average number of water molecules in a cluster

Half-peak width (Hz)	Average number	Samples	Sources
130	14	Piped water	*Is Water Medicine or Poison* [74]
119	12	Reverse osmosis water	*Is Water Medicine or Poison* [74]
119	12	Rainwater	Taiwanese scholar [75]
118	12	Distilled water	Taiwanese scholar [75]
105	11	Well water	Taiwanese scholar [75]
90	10	Small clusters water	Japanese scholar [75]
84	9	Mineral water	*Is Water Medicine or Poison* [74]
77	8	Nature mineral water	*Is Water Medicine or Poison* [74]
70	8	Longevity water	Japanese scholar [75]
68	7	High-quality mineral water	*Is Water Medicine or Poison* [74]
66	7	Bama longevity water	*Is Water Medicine or Poison* [74]
63	7	Glacial water	*Is Water Medicine or Poison* [74]
60	7	Hydrotherapy water	Japanese scholar [75]
54	6	Dag Ogawa glacier spring water	Chinese scholar [75]
53	6	Deep ocean health water	*Is Water Medicine or Poison* [74]
45	5	Fresh water from the local village	Chinese Academy of Medical Sciences [75]

### Application Examples of the Variation of Dissolved Oxygen in Different Water

To analyze the state of different water, five water samples were tested: small clusters water, purified water, deionized water, distilled water, and rainwater. Among them, small clusters water was produced with electromagnetic fields of diamond electrodes in the laboratory, purified water was obtained through a laboratory grade water purifier by Canature (China, Model JYEC-30), deionized water was prepared by AEMD platform of Shanghai Jiao Tong University, distilled water was obtained by a water distiller by Fuyao (China, model AR8406) in the laboratory, and rainwater was collected systematically in Shanghai. Five different water samples were placed in a 500 mL three-neck flask in turn, and the original gas in the flask was extracted in advance for 60 min by the jet pump, as a result, the dissolved oxygen in each water sample reached a low value and remained stable. Oxygen was then introduced into the water samples at a flow rate of 10 sccm until the dissolved oxygen in water reached a stable saturation concentration. After continuous oxygen introduction for 60 min, oxygen supply was stopped to allow the concentration of dissolved oxygen in the water samples to decrease naturally.

**Fig. 6:**
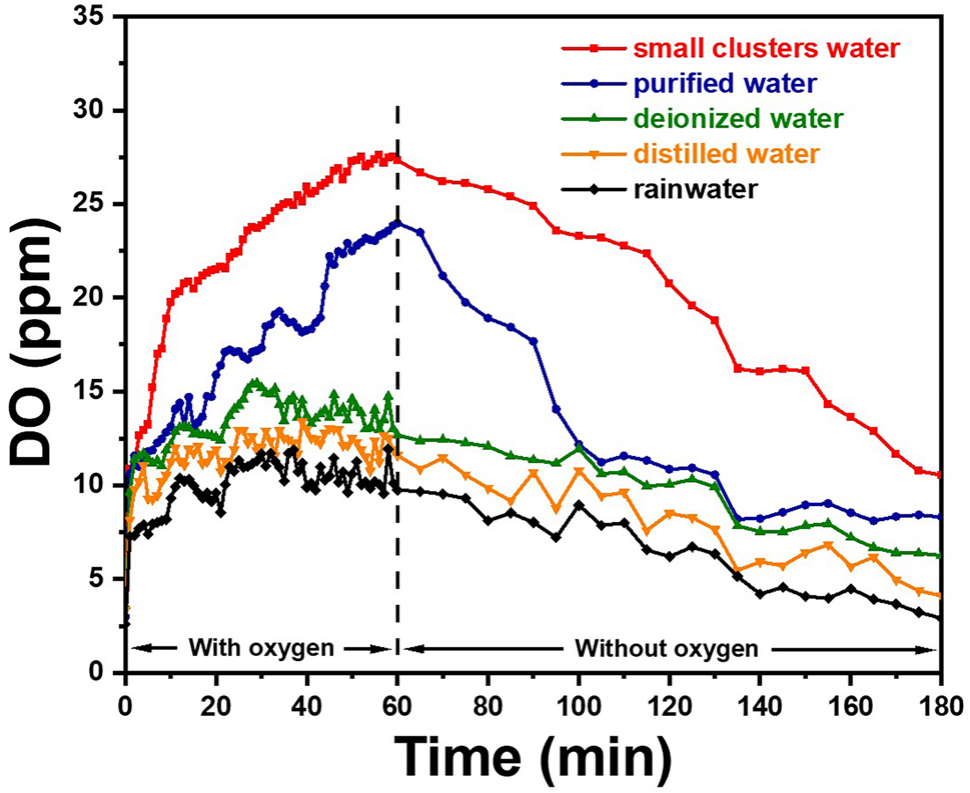
Variation of dissolved oxygen in five types of water samples under the condition of oxygen introduction (the first 60 min) and after the cessation of oxygen introduction (after 60 min)

The variations of the concentration of dissolved oxygen in each water sample were recorded during the experiment and the results are shown in Fig. 6. Overall, the concentration of dissolved oxygen decreased in sequence from small clusters water, purified water, deionized water, distilled water, to rainwater. Among them, small clusters water maintained a higher concentration of dissolved oxygen due to the treatment with diamond electrodes, exhibiting a stronger absorption capacity for oxygen. Moreover, its concentration of dissolved oxygen also remained higher after the cessation of oxygen introduction accompanied by a higher final concentration. On the contrary, the absorption and retention ability was relatively poor for rainwater due to the mixture of various organic or other pollutant substances. Meanwhile, purified water, deionized water, and distilled water with a reduction in the content of ions or other substances were successively purer, and consequently a sequential decrease in their ability to absorb and retain oxygen was observed.

**Fig. 7:**
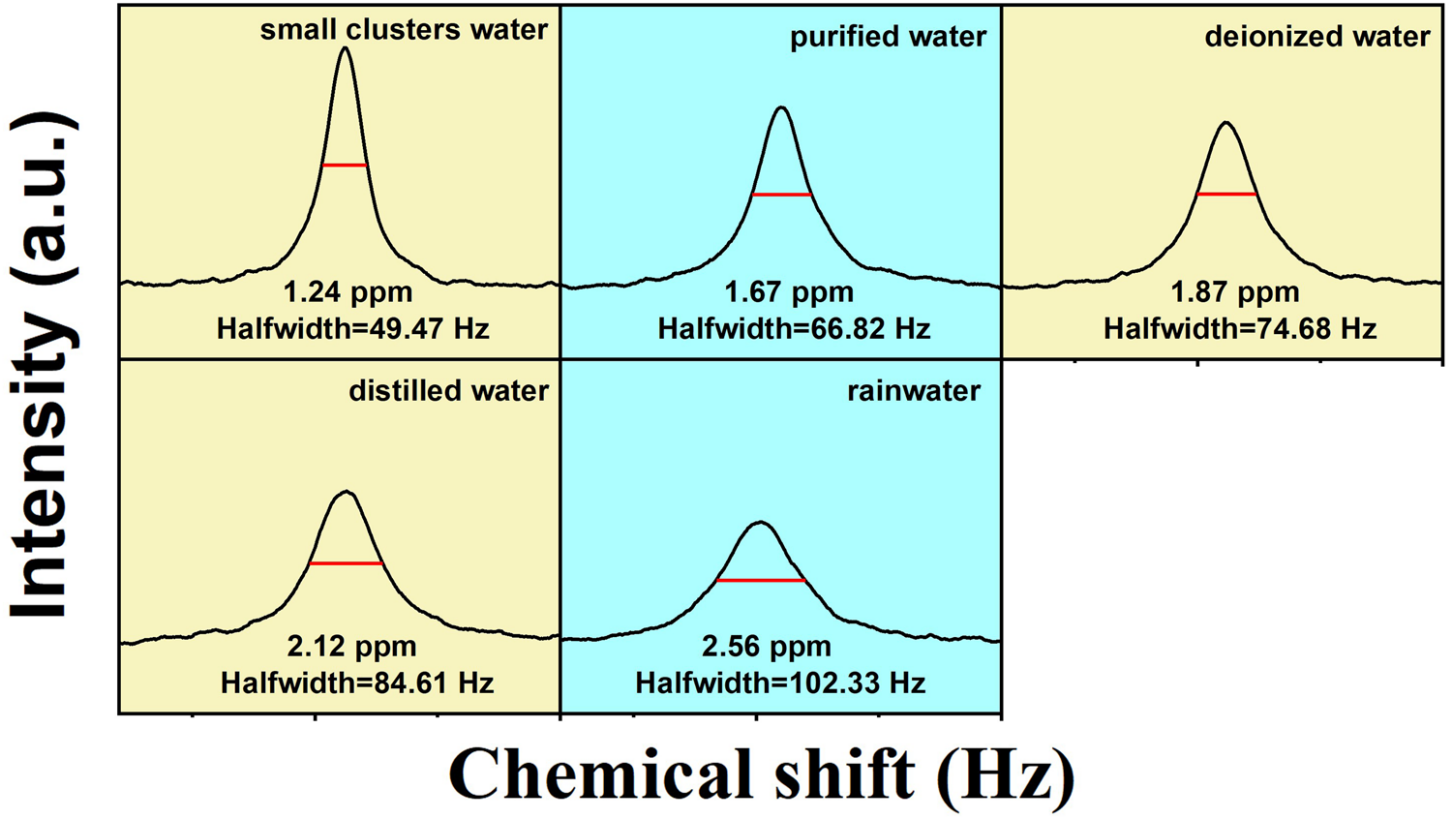
^17^O nuclear magnetic resonance spectra of five water samples

To further analyze the basic mechanism behind the varying capacities of absorbing and retaining oxygen of five types of water and explore the structures of different water, ^17^O NMR spectroscopy were performed and the results are shown in Fig. 7. It can be found that the ^17^O NMR half-peak width of each water sample increased successively from small clusters water, purified water, deionized water, distilled water to rainwater, meaning that the size of water clusters increased correspondingly. This was consistent with the pattern of variation in dissolved oxygen in Fig. 6. As an application example, the average sizes of water clusters in five water samples were calculated according to the summarized semi-empirical formula (1), and the results are listed in Table 2. It could be observed that the lower the NMR half-peak width, the smaller the average number of water molecules in a cluster, and the higher the concentration of dissolved oxygen. This result provided a theoretical basis and insights for improving the concentration of dissolved oxygen in practical applications.

**Table 2:** Results of ^17^O nuclear magnetic resonance half-peak width and the average number of water molecules in a cluster for five water samples

Water samples	Small clusters water	Purified water	Deionized water	Distilled water	Rainwater
Half-peak width (Hz)	49.47	66.82	74.68	84.61	102.33
Average number	5.64	7.32	8.08	9.04	10.75

In summary, molecular dynamics simulations of water clusters were performed, and the results confirmed that dissolved oxygen molecules can only stably exist at the interfaces among water clusters driven by free energy. This finding explained how oxygen molecules from the air could rapidly dissolve into water or how dissolved oxygen molecules could swiftly effuse to the surface of the water. Moreover, as the size of water clusters decreased, their surface area and the area of interfaces increased, leading to a higher saturation concentration of dissolved oxygen. Simultaneously, dissolved oxygen molecules seemed to be able to isolate water clusters and stabilize the water structure, indicating that the presence of dissolved oxygen molecules in the interfaces among water clusters played a role in stabilizing the equilibrium water structure.

## Conclusion

The existence forms and patterns of dissolved oxygen molecules in water has always been a significant topic in the field of water science research, making the study of the state and mechanism of oxygen in water of critical importance. In this paper, the relationship between water clusters and dissolved oxygen was analyzed. By dissolving different concentration of oxygen into distilled water and measuring the corresponding size of water clusters by ^17^O NMR technology, we found a clear negative correlation between the size of water clusters and the concentration of dissolved oxygen. This implied that smaller clusters water could absorb more oxygen molecules, allowing a higher value of saturated concentration of dissolved oxygen in water. We supposed this is because oxygen molecules are primarily existed at the surfaces or interfaces of water clusters and can rapidly traverse the gas–liquid interface through vibration and interfacial movement among water clusters. Further, simulations by molecular dynamics model of water molecules were performed to verify that dissolved oxygen molecules tend to exist at the surface interfaces of water clusters. Also, according to the data from literatures, we derived a semi-empirical formula relating the average number of water molecules in a cluster to ^17^O NMR half-peak width: *n* (average number) = 0.1 W (half-peak width) + 0.85, demonstrating an approximate linear relationship. As an application example, five types of water samples (small clusters water, purified water, deionized water, distilled water, and rainwater) were utilized for comparative analysis, and it was discovered that their saturated concentration of dissolved oxygen also exhibited a correlation with the size of water clusters. This further confirmed the influence of the size of water clusters on the concentration of dissolved oxygen and provided an important scientific phenomenon and theoretical basis for understanding the existence forms and patterns of dissolved oxygen in water, the balance and stability of water structure, and the methods to improve the concentration of dissolved oxygen in water. In summary, our research reveals that the distribution and concentration of dissolved oxygen depend not only on the partial pressure of oxygen and water temperature but are also significantly influenced by the size of water clusters. This has important scientific and practical significance for understanding and improving the concentration of dissolved oxygen in water.
